# Expression of Concern: Over-expression of microRNA-758 inhibited proliferation, migration, invasion and promoted apoptosis of non-small cell lung cancer cells by negative regulating HMGB

**DOI:** 10.1042/BSR20180855_EOC

**Published:** 2025-10-09

**Authors:** Guo-Hua Zhou, Yi-Yu Lu, Jing-Lian Xie, Zi-Kun Gao, Xiao-Bo Wu, Wei-Shen Yao, Wei-Guang Gu

**Affiliations:** 1 Department of Thoracic Surgery, Nanhai Hospital of Southern Medical University (People’s Hospital of Nanhai District), Foshan 528244, China; 2 Department of Oncology, Nanhai Hospital of Southern Medical University (People’s Hospital of Nanhai District), Foshan 528244, China

*Bioscience Reports* has been made aware of potential issues surrounding the scientific validity of this paper and hence is issuing an Expression of Concern to notify readers whilst the Editorial Office investigates. Concerns have been raised regarding the stylisation and appearance of the Western Blots, as well as the acknowledgment statement, which follows a pattern frequently seen in manuscripts where authorship is of concern. The authors have been contacted for comment and as of yet have not responded or provided the requested raw data.

